# Disfluencies in Public and Private Speech

**DOI:** 10.1177/00238309251380518

**Published:** 2025-11-10

**Authors:** Darinka Verdonik, Peter Rupnik, Nikola Ljubešić

**Affiliations:** Faculty of Electrical Engineering and Computer Science, University of Maribor, Slovenia; Jožef Stefan Institute, Slovenia

**Keywords:** Formal speech, spontaneous speech, interactional context, disfluency classification

## Abstract

This study investigates how speakers adapt their use of disfluencies in public versus private speech settings. Existing studies suggest systematic differences in disfluency rates, depending on who we are communicating with, how interactive the communication is, how difficult the topic is, whether the interaction is broadcast or not, and whether the speech is pre-scripted or not. We aim to improve this understanding through analysis in the Slovenian language, using data from the Training Corpus of Spoken Slovenian ROG-Artur. We investigate whether quantitative differences in the use of disfluency exist between private and public speech, and aim to explain these differences by investigating the relationship between disfluency functions and the physical, social, cognitive, and other factors influencing communication behavior. Our results revealed significant differences in disfluency patterns: disfluencies, in general, are more frequent in private speech, whereas filled pauses, unrepaired pronunciations and blocks, are more common in public speech. We group disfluency functions into two general categories. In contextual analysis, we interpret that speakers reduce disfluencies in public speech due to its high relevance, formal expectations, partial pre-scripting, time constraints and advanced speaker skills, while the higher frequency of filled pauses, unrepaired pronunciations and blocks in public speech reflect the impact of longer dialog turns, time constraints and emotional stress. The findings of this study should be interpreted with caution, given the interpretative nature of qualitative analysis and the potential confounding effect of the involvement of different speakers in the public and private speech samples.

## 1 Introduction

Speech is produced in real time as the speaker speaks. During this process, the speaker may change the intended course of the utterance (e.g., restart), correct an error (e.g., self-repair), slow down the rate of speech (e.g., repetition, filled pause), or the like. These are all common speech production strategies, and do not cause difficulties in communication; on the contrary, they can make it more effective (Clark, 2002). However, these phenomena have been given the term disfluencies, which has a somewhat negative connotation. Disfluencies have been defined as “phenomena that interrupt the flow of speech and do not add propositional content to an utterance” (Fox Tree, 1995, p. 709). Despite their negative connotation in the past, scholars warn that it is questionable to consider features of spoken discourse as “deviant” and “irregular” when they occur with a frequency that borders on regularity in “normal” native speaker use of the language (Stenström & Svartvik, 1994, p. 242). Many have argued that, in everyday communication, they do not occur merely in contexts of speech trouble, but they also serve other—interactional—functions (Clark, 2002; Crible, 2018; Kosmala, 2024; Schegloff, 2010; T. J. Taylor, 1997; Tottie, 2014, 2016, 2019). Clark (2002, p. 5) suggested that speakers design most forms of disfluency as communicative acts to coordinate their speech acts with their listeners, that is, speakers try to synchronize their vocalizations with their listeners’ attention, or they try to synchronize the presentation of each expression with their listeners’ analysis of those expressions. Schegloff (2010) argued that filled pauses can, in some usages, serve functions such as marking the start of an interaction, signaling a forthcoming mispreferred response or sequence, or indicating an attempt to re-close an unresolved sequence. Tottie (2016) showed that filled pauses are not merely signs of hesitation, but function as pragmatic markers comparable to *well*, *you know*, and *I mean*. Based on the analyses of their usage in American journalistic writing (Tottie, 2019) she found that, in written text, they are used to expresses the writers’ attitude to the content, while, in speech, they are used to help speakers plan their upcoming speech. Götz (2013) pointed out that disfluencies can be regarded as part of fluency phenomena, and can be studied from a speaker’s or listener’s perspective. Researchers such as Crible (2018) adopted Götz’s notion of fluencemes, and argued that they “are not necessarily problematic, but rather reflect some cooperative (even listener-oriented) search for the optimal utterance” (Crible, 2018, p. 22). All this suggests that examining disfluencies still offers valuable insights into the communicative processes, and the physical, cognitive, social, and other contextual factors that shape spoken interaction.

As noted by McDougall and Duckworth (2017), the majority of research on disfluency is interested in the role of disfluency phenomena in speech planning and production (e.g., research such as Brennan & Schober, 2001; Corley et al., 2007; Fox Tree, 2001; Fraundorf & Watson, 2011; Levelt, 1983; MacGregor et al., 2010). A vast amount of research can be found on disfluencies in speech disorders (e.g., Penttilä et al., 2019), language teaching and learning (e.g., Frobenius, 2022), automatic disfluency detection (e.g., Passali et al., 2024). Furthermore, researchers have proved that the use of disfluencies is speaker specific (Horvath, 2007; Shriberg, 2001; Taschenberger et al., 2019), where the greatest levels of speaker-specificity according to discriminant analysis are the filled pauses *er* and *erm*, prolongations and word repetitions (McDougall & Duckworth, 2017). Disfluencies tend to be more common in longer sentence units (Moniz et al., 2014; Oviatt, 1994), before words that are less predictable, and therefore more cognitively demanding (Sen, 2020), and they are more likely to appear early in the phrase (Shriberg, 2001). However, Kosmala and Morgenstern (2019) found that filled pauses may be an exception, as, in their data, they found little relation between the rate of fillers and the perceived difficulty of the questions. The majority of research on disfluencies relies on quantitative research. Kosmala (2024, p. 32) warned that there is a lack of qualitative research which would illuminate their contextual, situational and interpersonal dimension.

Some researchers investigated the relationship between demographic factors and disfluencies in speech. The research of Shriberg (2001) and Bortfeld et al. (2001) indicated that men make more disfluencies per word than women do, due mainly to a higher rate of filled pauses (Bortfeld et al., 2001). Engelhardt and Markostamou (2024) found that younger adults produced more *ums* and fewer repetitions compared to older adults, while Taschenberger et al. (2019) found that age does not play a great role in the use of disfluencies. However, Bortfeld et al. (2001) found that only speakers over the age of 63 produced higher disfluency rates than middle-aged and younger speakers, while there was no difference between the younger and middle-aged groups.

Our study aims to contribute to our understanding of how speakers adapt their use of disfluencies to an interactional context. Some sporadic research suggests that there are systematic differences in disfluency rates, depending on who we communicate with—a speaker or a computer (Oviatt, 1994; Shriberg, 2001), how interactive the communication is (Moniz et al., 2014; Oviatt, 1994), how difficult the topic is (Bortfeld et al., 2001), or whether the interaction is broadcast or not (Crible, 2018). Our study seeks to add to this body of research by investigating how disfluency usage differs between public and private speech. Compared to previous research, the advantages of our study are that we (1) Prepared a balanced data sample, covering different genres and a high number of speakers from various demographic groups, (2) We developed a corpus-driven categorization of disfluencies, and compared the frequencies between various disfluency types, (3) We did not perform solely a quantitative, but also a detailed qualitative analysis of disfluency usage in public and private speech. Although qualitative analysis inevitably involves a degree of subjectivity, it remains essential for explaining language use, (4) We included a systematic analysis of contextual features, considering physical, social, cognitive and other factors that influence the communication behavior in our public and private data, and (5) We conducted our research on Slovenian, thus extending the existing research on disfluencies by exploring a new language. Previous research on disfluency in the Slovenian language has been unsystematic or partial, and based on very limited data (Verdonik, 2006).

The remainder of this article is structured as follows. Section 2 offers an overview of related work, while Section 3 details the data, the disfluency annotation scheme, and the employed analytical procedures. Section 4 presents the findings on disfluency frequencies, their functions, and the influence of contextual features on disfluency usage. Sections 5 and 6 provide the discussion and conclusions.

## 2 Related works

Comprehensive reviews of research on disfluencies can be found, for example, in (Gotz, 2013; Kosmala, 2024 and, others). In this section, we focus specifically on research findings that are directly relevant to the central topic of this study. We begin by reviewing previous findings on the influence of interactional settings on disfluency use. The reviewed literature is organized into five thematic categories: (1) the influence of the circumstances and entities we communicate with; (2) the degree of interactivity in communication; (3) the complexity or cognitive demands of the situational context; (4) the distinction between broadcast and non-broadcast speech; and (5) the level of speech spontaneity. In Section 2.2, we review existing classifications of disfluencies that provide the framework for the classification applied in our study.

### 2.1 Influence of interactional setting on disfluencies` use

First, based on the past research, we can assume that fluency of speech is adapted to the circumstances and entities we communicate with. There is strong evidence that disfluencies are more frequent when people communicate with other humans than when they communicate with computers. In her research, Shriberg (2001), used three corpora of American English: The Switchboard corpus, containing telephone conversations between strangers who chose topics from a predefined set; the AMEX corpus, which consists of air travel planning dialogs between travel agents and callers making actual travel plans; and the ATIS corpus, where the speakers were arranging hypothetical travel scenarios by interacting with a computer. Based on these data Shriberg (2001) found that a conversational partner, that is, a human versus a computer, is an essential factor in the disfluency rate, as well as in the disfluency distribution. However, she also noted that the “suppressed rates of disfluencies in human-computer dialogue for ATIS is also partly due to the presence of a push-to-talk mechanism” (Shriberg, 2001, p. 155). Nevertheless, the findings of Oviatt (1994) were similar—disfluency rates in human-computer interaction (volunteers using a service transaction system which assisted users with tasks such as conference registration, car rental, etc.) were substantially lower than in comparable human-human speech. Systems for human-computer communication have advanced significantly since these early studies, and scholars are now experimenting to add disfluencies into computer speech automatically. Therefore, it is questionable whether the same behavior from users of such systems is to be expected nowadays. Furthermore, Bortfeld et al. (2001) proposed that one possible conclusion was that speakers put more effort into communication with machines, and “speakers who try to take more care with their speech may succeed in producing more fluent utterances” (Bortfeld et al., 2001, p. 143).

Second, there are reports that disfluencies are more frequent in interactive communication. Oviatt (1994) compared disfluencies in two-person telephone conversations, a three-person interpreted telephone conversation with a professional interpreter intermediating, two-party face-to-face dialogs, and single-party monologues into an audiotape machine. She reported that disfluencies tended to be more frequent in telephone conversations than in face-to-face dialogs, and the least frequent in noninteractive monologs. Moniz et al. (2014) explored speaking style effects in the production of disfluencies in university lectures and map-task dialogs. Their data consisted of a university lectures corpus and a corpus of task-oriented dialogs. The data contrasts in the degree of interactivity (monologic lectures, dialogic interaction between speakers who tried to negotiate the route on the map), as well as formality (lectures can be considered as a formal communication, while a one-to-one task-oriented setting may be considered as non-formal). They found that dialogs had a higher percentage of disfluencies than lectures. The distributional patterns also differed. While filled pauses were the most common in both corpora, there were more repetitions and fragments in dialogs, and more silent pauses and complex sequences of disfluencies in lectures. The authors offered an explanation based on time constraints: “/Teachers have more time to edit their speech, displaying strategies associated with more careful word choice and speech planning, whereas dialogue participants had stricter time constraints” (Moniz et al., 2014, p. 33). However, the findings of Kosmala (2024) were the opposite. Her study examined the use of disfluencies in two distinct interactional contexts: formal classroom presentations delivered before an audience, and face-to-face casual dyadic conversations between friends. Despite a limited data set of 30 min of recordings for each context, it offered the advantage of using the same speakers across both data sets, to minimize the influence of individual speaker variation on disfluency patterns. Combining qualitative and quantitative methods, she found more frequent disfluencies in classroom presentations, with longer silent pauses, more non-lexical sounds and filled pauses. Notably, individual differences sometimes contradicted general trends. Her findings highlighted the need for caution when generalizing disfluency patterns from small samples, emphasizing the influence of personal speaking styles on fluency.

Third, Bortfeld et al. (2001) provided evidence that disfluency rates increased in more challenging situational contexts. They examined how speakers behaved in familiar versus unfamiliar domains and across different speaking roles. Their data were task-oriented, involving 48 pairs of speakers engaged in matching two sets of picture cards: photographs of children (familiar domain) and abstract geometric forms (unfamiliar domain). During the tasks, the speakers alternated roles as coordinator (director) and participant (matcher). The results indicated higher disfluency rates in the unfamiliar domain, due primarily to repeats and restarts. Similarly, speakers in the coordinator (director) role produced more disfluencies, due largely to filled pauses and restarts. These results aligned with the findings of Schachter et al. (1991), who hypothesized that the greater the number of options, the more likely a speaker is to say “uh..” Since academic disciplines vary in the extent to which their subject matter and mode of thought require speakers to choose among options, they suggested that the more formal, structured and factual a discipline, the fewer the options, and, consequently, the fewer filled pauses. Their data, drawn from the speeches of 45 lecturers across 10 departments representing the Natural and Social Sciences and the Humanities, supported this claim.

Fourth, Crible (2018) found that broadcast versus non-broadcast interaction affected the use of disfluencies. She used an English and French corpus of face-to-face interviews (17,000 and 18,000 tokens respectively) and radio interviews (about 4,000 tokens for each language). Her results showed that, overall, there were more disfluencies in non-broadcast interviews than in broadcast interviews. Comparing specific types of disfluencies, her results showed that the only type of disfluency that is consistently more frequent in both languages in broadcast discourse was identical repetitions. She explained this as a possible result of a specific radio style (Crible, 2018, p. 134), while the remaining disfluencies tended to be more frequent in non-broadcast interviews, or showed no significant difference. This single study on how the broadcast or public speech affects the use of disfluencies compared to non-broadcast or non-public speech was based on a single genre, that is, interviews, and a rather small amount of data, with no information on the number of speakers included. As stated in the Introduction, the studies have shown that the use of disfluencies is speaker specific; consequently, the variation between both speech types may be partly speaker specific when the number of speakers is small.

Fifth, the study of Tonetti Tübben and Landert (2022) suggested that the use of disfluencies depends on the level of speech spontaneity. They analyzed the frequency and functions of “uh” and “um” across three genres: spontaneous conversations, scripted dialogs in television series, and dialogs in improvised theater. They found that filled pauses (referred to as planners in their study) were significantly more frequent in spontaneous speech, such as conversations and improvised theater, compared to scripted dialogs. Nevertheless, these pauses were present in all genres, and fulfilled all the four functions identified in their analysis.

In summary, the reviewed studies provide compelling evidence that the use and distribution of disfluencies are tied closely to a range of contextual factors. First, the research indicated that fluency is adapted to the communicative environment, with speakers displaying significantly lower disfluency rates when interacting with computers compared to human interlocutors—though these findings may need to be revisited in light of advancements in human-computer interaction. Second, the degree of interactivity appears to play a significant role, with more disfluencies typically observed in interactive dialog than in monologic or formal settings; however, exceptions, such as Kosmala’s (2024) findings, highlighted the importance of large data sets. Third, situational complexity—whether due to unfamiliar content or role-based demands—has been shown to increase disfluency rates, supporting the view that cognitive load has an important effect on the fluency of speech. Fourth, the communicative context, particularly the distinction between broadcast and non-broadcast settings, influences the frequency and type of disfluencies, though current evidence is based on limited and genre-specific data sets. Finally, the level of speech spontaneity correlates strongly with disfluency usage, with spontaneous and improvised speech yielding a higher frequency and functional diversity of filled pauses than scripted language. Taken together, these findings reinforce the view that disfluencies are not merely random disruptions in speech, but are shaped by interactional, cognitive, and situational factors.

### 2.2 Disfluencies` classification

The classification of disfluencies is undertaken most commonly for the purpose of data annotation. One of the earliest classifications (Maclay & Osgood, 1959), identified four hesitation types: repeats, false starts, filled pauses, and unfilled pauses. Subsequent classifications offered greater detail. An influential scheme was proposed by Shriberg (1994), who suggested that the main types of disfluencies included filled pauses, repetitions, deletions, substitutions, insertions, and articulation errors (Shriberg, 2001). The Switchboard Corpus annotation (Meteer & Taylor, 1995) included tags for incomplete utterances, various types of restarts, and non-sentence elements, such as fillers, editing terms, discourse markers, conjunctions, and asides. The Penn Treebank scheme (A. Taylor et al., 2003) aligned closely with the Switchboard Corpus annotation system. Strassel (2003) introduced a Simple Metadata Annotation Specification for the Linguistic Data Consortium, distinguishing between fillers (filled pauses, discourse markers, explicit editing terms, and asides or parentheticals) and edit disfluencies (repetitions, revisions, restarts, and complex disfluencies); the former could be annotated based on their structure as well. Besser and Alexandersson (2007) developed an annotation scheme for the AMI corpus. They categorized disfluencies into three main types: (1) uncorrected strings, subdivided further into uncorrected mistake, omission or order, (2) deletable elements, including delays, disruptions, slips of the tongue, and editing terms; and (3) revisions, which encompassed various types of self-repairs. More recent schemes (Crible et al., 2022) distinguished lengthenings, blocks, filled pauses, silent pauses, discourse markers, self-interruptions, modifications, and repetitions at the first level, with several subtypes defined for each category at the second level; while Kosmala (2024) distinguished vocal disfluencies (prolongation, unfilled pause, filled pause), morpho-syntactic disfluencies (self-repairs, self-interruptions, truncated words, identical repetitions), and peripheral disfluencies (explicit editing terms, non-lexical sounds).

As is evident from the overview above, certain disfluency categories are present consistently across all classifications, including filled pauses, repetitions, and self-repairs (also referred to as false starts, restarts, revisions, or modifications). The latter is often subdivided further into various subcategories, such as deletions, substitutions, insertions, or revisions and restarts. Other disfluency categories, such as silent pauses, lengthening, and discourse markers and/or editing terms, appear in some classification schemes, but not in all. In addition, a few disfluency categories are mentioned only occasionally, such as articulation errors/slips of the tongue, blocks, incomplete utterances, asides and complex disfluencies.

The research presented in Section 2.1 focuses typically on the most frequent disfluency categories. Filled pauses are generally identified as the most frequent, and are notable for their specific discourse functions (Bortfeld et al., 2001). Self-repairs of various types (referred to as corrections, restarts, or similar) constitute the second most frequent category, followed closely by repetitions. Silent pauses and lengthening are often excluded from analyses, as their classification as disfluencies can vary significantly between annotators (Bortfeld et al., 2001). While discourse markers can also occur frequently, they are found to serve numerous discourse functions (Crible & Degand, 2019) and are not regarded typically as standard disfluencies.

## 3 Data and methodology

The aim of our study was to investigate how disfluency usage differs between public and private speech. We set the following research questions:

*Research Question 1 (RQ1).* Are there quantitative differences in the use of disfluencies between private and public speech?
*Research Question 2 (RQ2).* Do different types of disfluencies serve different communicative functions?
*Research Question 3 (RQ3).* Is there a meaningful relationship between physical, social, cognitive and other factors that influence speakers’ communication behavior and the functions of disfluencies that might explain the quantitative differences between private and public speech?

We collected and annotated data, and conducted both quantitative and qualitative analyses, as outlined below.

### 3.1 Data

This study is based on the Training Corpus of Spoken Slovenian ROG-Artur (Verdonik, Dobrovoljc, et al., 2024). The data originally formed part of the Artur corpus (Verdonik, Bizjak, et al., 2024). 60% of the ROG-Artur corpus comprises samples from public events, provided by the Slovenian Press Agency, a local radio station (Radio Štajerski val), and the Slovenian National Assembly. 40% of the data consists of private samples provided by students, who were recruited to record individuals in their local environments. The speakers were asked to engage in conversations with friends (always in pairs), or to provide explanations and descriptions about topics such as their trips, hobbies, favorite films or books, meals they prepare, and so on. The public and private speech samples did not involve the same set of speakers; thus, individual speaker characteristics may have influenced the results to some extent. To mitigate this effect, a relatively large number of speakers was included in each data set—47 in the public speech sample and 28 in the private speech sample. The samples included in the ROG corpus are not entire recordings or complete speech events, but, rather, randomly selected excerpts ranging from 3 to 6 min in duration. Table 1 presents the structure of the corpus, while Tables 2 and 3 provide details on the speakers in both data sets.

**Table 1.** ROG-Artur Structure.

Discourse type	Speech event	Number of samples	Number of words
Public speech	Online event	7	5,787
	Round table	6	5,043
	Interview	6	5,136
	Press conference	3	2,478
	Speech at the event	1	732
	Session of the National Assembly	6	4,295
	**Public—total**	**29**	**23,471**
Private speech	Dialog	14	5,770
	Monolog	14	9,760
	**Private—total**	**28**	**15,530**

**Table 2.** ROG-Artur Speakers—Public Speech.

		Number of speakers	Number of words
Gender	Male	21	10,523
	Female	28	12,948
Age	18 to 34	0	0
	30 to 59	26	12,647
	60+	8	3,558
	Unspecified	14	7,266
Total		**47**	**23,471**

**Table 3.** ROG-Artur Speakers—Private Speech.

		Number of speakers	Number of words
Gender	Male	17	9,609
	Female	11	5,921
Age	18 to 34	11	6,779
	30 to 59	10	5,734
	60+	7	3,017
	Unspecified	0	0
Total		**28**	**15,530**

### 3.2 Disfluency categories and annotation

Our approach to disfluency annotation was corpus-driven (Tognini-Bonelli, 2001), meaning that disfluency categories were identified directly from the corpus without relying on preconceived theoretical frameworks. We annotated as disfluencies a broad range of phenomena that, in specific contexts, can be recognized as instances where the processes of speech production are revealed, whether at the level of sound formation and articulation, or syntax structure and meaning formation. Our aim was to develop a simple and robust annotation scheme.

The annotation process involved four steps: (1) A disfluency expert reviewed the data thoroughly, and developed an initial annotation scheme based on the relevant literature (see Section 2.2); (2) The initial scheme was tested on several data samples and refined; (3) A single student was trained to annotate the entire data set; and (4) The expert reviewed all the annotations made by the student meticulously, finalized the annotation scheme as detailed below, and adjusted all the annotations to align with the final scheme. The annotation was performed using the EXMARaLDA Partitur Editor (Schmidt & Wörner, 2014).

In the final scheme, a primary distinction was made between disfluencies related to the content of speech and those related to the manner of delivery. The former were categorized as verbal disfluencies, while the latter were categorized as vocal disfluencies, a distinction also noted by Kosmala (2024, pp. 86–90). It was observed that vocal disfluencies, such as filled pauses, can occur simultaneously with verbal disfluencies. To account for this, vocal and verbal disfluencies were annotated on separate, parallel tiers. For instance, both the self-repair and the filled pause were annotated when a filled pause occurred during the interregnum phase of a self-repair. In addition, a third annotation tier was introduced specifically for self-repairs, marking the reparandum, the interregnum (if present), and the repair sequence.

A detailed categorization and description of all the disfluency categories included in the final annotation scheme are presented in Tables 4 and 5.

**Table 4.** Verbal Disfluencies.

Category	Subcategory	Description
Repetition		The speaker repeats a word or phrase two or more times consecutively, but not for emphasis. The repetition may or may not be accompanied by a silent pause, a filled pause, or lengthening. Examples: *da tisti ki ki* “so the one that that”; *bolj ali manj v eem v* “*more or less in uhm in*”; *ko igram glasbo ko igram glasbo pozabim na vse probleme* “*when I play music, when I play music, I forget all the problems*”
Self-repair	Pronunciation	The speaker re-articulates a word before it has been pronounced fully. Examples: *b() bo* “w”() will; *da() določene* “spi() specific”
	Lexicogrammar	The speaker revises their speech by replacing, adding, and/or omitting one or more words fully or partially. Examples: *nekam—nikoli* “somewhere —never”; *da človek ne poškodu()—da robot ne poškoduje* that human does not ha()—that robot does not harm’; *v tej—v večini teh* “in these—in most of these”
	Restart	The speaker abandons the initiated structure and restarts the sentence and/or thought. The initial structure is neither completed nor revised, but is discarded entirely, with the speaker proceeding to express the content using a new structure, often rephrased differently. Examples: *pa smešno je ker imajo—recimo enkrat sva hotela iti gledat* “and it’s funny because they have—for example once we wanted to go watching; *glih je š()—letelo je mimo tega gorovja* “exactly pass()—it flew right above these mountains”
	Complex	Multiple disfluencies may occur consecutively within a segment of speech, or the speaker might alter the planned sentence structure without correcting all the previously spoken parts that are structurally related to the revision fully, or without completing the repair sequence. Such cases, along with other self-repairs that do not align with the previously defined categories, are annotated as complex disfluencies. Example: *to naše področje medijsko no medijsko je zelo široko področje novinarsko področje založniško bom tako rekla novinarsko zaro() založniško* “this our field of media, well media is a very broad field, the field of journalism, the field of publishing. I’ll put it this way. journalist publi() publishing.”
Commentary	Disfluency marker	A discourse marker that serves primarily to signal difficulties in speech production. Common examples of such markers in our data include: *ne vem* “I don’t know”, *bi rekel* “I would say”, *se pravi* “so to speak”, *mislim* “I mean”
	Disfluency comment	Expressions or sentences in a speech that comment on difficulties in speech production are annotated as disfluency comments. They differ from disfluency markers in that their meaning is conveyed propositionally. Examples: *se opravičujem* “I appologize”; *kako se reče* “how do you say”; *kaj sem zdaj mislila reči* “what was I about to say.”
Unrepaired	Pronunciation	The speaker mispronounces a word by using incorrect sounds and does not correct the pronunciation. Example: *zuženih (združenih)* “nited (united).”
	Structure	A syntactic unit, such as a sentence, contains a mismatch that does not follow an established pattern, even in conversation or dialect, but, instead, represents an error or an unexpected change in the anticipated continuation of the sentence. Example: *hkrati se pa pojavlja tudi nove bolezni* “at the same time it appears new sicknesses”
Abandoned		The speaker does not complete the structure he started, but instead relinquishes the turn to the interlocutor. This may occur when the speaker is searching for a word and the interlocutor takes over, when the speaker attempts to take the turn but is unsuccessful, or when the structure is deliberately left unfinished because a response from the interlocutor is anticipated. Such instances are annotated as abandoned.

**Table 5.** Vocal Disfluencies.

Category	Description
Lengthening	A specific phoneme or syllable within a word may be extended beyond its typical duration, or an entire word may be articulated more slowly with added prolongation. If such prolongation does not signal the end of a syntactic-semantic unit or serve as a rhetorical device, it can be classified as a disfluency. The annotation of this phenomenon relies largely on auditory perception, and the annotator’s subjective judgment as to whether the prolongation indicates that the speaker is pausing to gain time for speech formulation or not.
Silent pause	A silent pause in speech may or may not indicate disfluency. Speakers often use pauses between sentences or statements for rhetorical purposes or other reasons, making the distinction between these functions unclear and difficult to define. Generally, pauses occurring within a semantic-syntactic unit or statement are more likely to be considered disfluencies, while those between statements or sentences are classified as such less frequently. Furthermore, in slow speech, only notably long pauses are marked typically, whereas, in fast speech, even brief pauses can be perceived as disfluencies.
Filled pause	In the Slovenian speech, the mid-central vowel /Ə/ is used frequently, and less commonly the voiced consonants /m/ or /n/, in a manner perceived as fillers or referred to as gap fillers. Such sounds are annotated consistently as filled pauses without exception.
Non- linguistic sound	The speaker clears their throat, coughs, or produces a similar sound, indicating a potential issue with the speech apparatus.
Block	The speaker does not pronounce a particular word entirely fluently. There may be hesitation at the beginning, or a gap may occur during the pronunciation. However, the word is produced in the correct form ultimately, and the speaker does not restart the pronunciation.

### 3.3 Analytical procedure

The analytical procedure follows a sequential approach, addressing the first, second and third research questions in order.

First, we measured the differences in disfluency usage between public and private speech quantitatively. To assess the statistical significance, we applied a chi-square test, and to measure effect size, we calculated the odds ratio. This simple effect size indicates, provided the frequencies are sufficiently high for reliable measurement, how much more likely a disfluency is to occur in one type of speech compared to the other.

Second, we conducted a detailed qualitative analysis of the functions associated with each disfluency type defined in Tables 4 and 5. Our approach was grounded in interactional sociolinguistics (Toomaneejinda & Saengboon, 2022). Using the EXMARaLDA Analysis and Concordance Tool EXAKT, we searched for all instances of specific disfluency types within our data set. This investigation extended beyond examining the transcription alone; we also listened carefully to the audio recordings to contextualize the disfluencies fully. This method enabled us to identify the potential functions that each disfluency type serves. Discourse features are inherently multifunctional, with their functions shaped by the specific contexts in which they occur. Due to the large volume of examples, the analysis focused on identifying the most typical and widely observable functions. Such an approach is in line with the theory of norms and exploitations (Hanks, 2013), according to which, linguistic forms acquire meaning through repeated, socially shared patterns of use (norms), while speakers can also manipulate these norms creatively (exploitations) to produce context-specific effects. By prioritizing the most recurrent functions, the analysis aims to capture the normative uses of discourse features, while acknowledging that less frequent, context-sensitive exploitations also exist and merit further investigation. Inevitably, this approach entails a certain level of subjectivity, given its reliance on the analyst’s interpretation of the data. To reduce this limitation, the analysis is cross-referenced with the findings and interpretations from previous research.

Third, we analyzed the physical, social, cognitive and other factors influencing speakers’ communication behavior, referred to as contextual features. Although we refer to the data set as a corpus, we are highly familiar with the data. All the recordings were listened to multiple times during the annotation process, and we had access to detailed contextual information for each recording, including the time, location, topic, occasion, and identity of the speakers. Drawing on van Dijk’s (1997) elements of context models, we defined the context of public and private speech in our data in relation to: (1) The scene; (2) Assigned relevance and importance; (3) Aims; (4) Conversational dynamics; (5) Positions and social relations; and (6) Social affiliations. We adopted van Dijk’s socio-cognitive approach to context (van Dijk, 1997, 2001, 2007), which highlighted the importance of cognitive perspectives in understanding the relationship between context and text. In this framework, context is viewed as a subjective mental construct shaped by the participants’ interpretations of the social environment. Consequently, we caution readers that our interpretations represent our own subjective perspective, albeit one that has been considered carefully and reflected upon critically multiple times.

Finally, the differences in disfluency frequencies were explained in relation to the observed functions of disfluency types and the contextual features of public and private speech.

## 4 Results

This section is organized so that the first, second, and third subsections correspond to the respective research questions.

### 4.1 Quantitative trends in disfluency type in public versus private speech

In the first section of the results, we tackled the first research question: Are there quantitative differences in the use of disfluencies between private and public speech? To answer this question in sufficient detail, we combined inferential and descriptive statistics.

For the inferential statistics we used the chi-square test for significance testing, and, in the case of significant results, the odds ratio for quantifying the effect size. To perform the chi-square test, we constructed a contingency matrix of speech-type dependent counts of positive and negative events. We considered each disfluency as a positive event, while the total number of words in the transcripts represented the overall number of events. The difference between the total word count and the number of positive events was taken as the number of negative events. Different types of disfluencies may occur on a single word, across multiple words, or between words; however, for consistency, we considered each word a potential instance of disfluency.

In the descriptive part of our quantitative results, we reported the number of occurrences of each specific disfluency type per 1,000 words. A tabular representation of the total occurrences of each disfluency type in public and private speech, along with their frequency per 1,000 words, is provided in Table 6.

**Table 6.** Absolute and Relative (per 1,000 Words) Counts of Disfluencies by Type of Disfluency, and by Speech Type (Public vs. Private).

	Public	Private	Public (1kw)	Private (1kw)
**Verbal**				
Self-repair Lexicogrammar	160	137	5.56	7.02
Repetition	79	87	2.74	4.46
Self-repair Pronunciation	84	48	2.92	2.46
Disfluency Marker	40	49	1.39	2.51
Unrepaired Pronunciation	60	20	2.08	1.02
Unrepaired Structure	43	32	1.49	1.64
Abandoned	14	54	0.49	2.77
Self-repair Complex	18	36	0.63	1.84
Self-repair Restart	11	30	0.38	1.54
**Vocal**				
Silent Pause	969	1,011	33.66	51.80
Filled Pause	1,213	659	42.13	33.77
Lengthening	709	588	24.63	30.13
Block	44	14	1.53	0.72
Non-linguistic Sound	13	12	0.45	0.61
**Overall**	3,457	2,777	120.08	142.29

Our first measurement was focused on the overall difference in the usage of disfluencies between public and private speech. Testing the null hypothesis, which states that there is no association between speech type (public vs. private) and the occurrence of disfluencies, we obtained χ²(*df* = 1, *N* = 48,305) = 50.86, *p* = 9.91e−13, with the odds ratio effect size of OR = 1.22. This result indicated an association between the speech type (public or private) and the frequency of disfluencies, with the odds of disfluencies occurring in private speech being 1.22 greater than in public speech.

We next focused on the verbal disfluencies, analyzing the trends for each disfluency type separately based on their frequency per 1,000 words. As shown in Table 6, two types of verbal disfluencies deviated from the general pattern: self-repair pronunciations and unrepaired pronunciations. Both phenomena occurred infrequently in our data, with fewer than 100 instances for each speech type. While no significant difference was found for self-repair pronunciations, χ²(*df* = 1, *N* = 48,305) = 0.74, *p* = .39, a significant difference was observed for unrepaired pronunciations, χ²(*df* = 1, *N* = 48,305) = 7.27, *p* = .007, with a notably high odds ratio of OR = 2.04. This result suggested that unrepaired pronunciations were twice as likely to occur in public speech compared to private speech.

We next turned to vocal disfluencies, analyzing trends by each disfluency type, to determine whether any deviated from the established general trend of disfluencies being more prominent in public speech. In Table 6 one frequent category stands out with an opposite trend: filled pauses. Given their high frequency, it is unsurprising that the difference is statistically significant, with χ²(*df* = 1, *N* = 48,305) = 21.63, *p* = 3.3e−06, and an odds ratio of OR = 1.26. Another category that deviated from the general trend was blocks, which occurred much less frequently than filled pauses. Despite the low number of occurrences in both types of speech, the difference was statistically significant, with χ²(*df* = 1, *N* = 48,305) = 5.27, *p* = .02, and an odds ratio of OR = 2.13.

We concluded this Section by calculating the chi-square test and odds ratio effect size for all disfluency categories that either followed the overall trend of being more frequent in private speech, or showed no statistically significant association with either type. The result was χ²(*df* = 1, *N* = 48,305) = 153.08, *p* = 3.68e−35, with an odds ratio of OR = 1.49. This indicated that, with the exception of unrepaired pronunciations, filled pauses, and blocks, most disfluencies are more likely to occur in private speech, with the odds being 1.5 times higher compared to public speech. Conversely, for the three aforementioned disfluency categories, the analysis yielded χ²(*df* = 1, *N* = 48,305) = 30.31, *p* = 3.68e−08, with an odds ratio of OR = 1.3, indicating that these disfluencies are 1.3 times more likely to occur in public speech than in private speech.

From these quantitative measurements, we can conclude that (1) Disfluencies overall are more associated with private speech, with an odds ratio of 1.22, and (2) Among these, unrepaired pronunciations, filled pauses and blocks are associated positively with public speech, with an odds ratio of 1.3.

### 4.2 Disfluencies’ functions

In the second section of the results, we tackled the second research question: Do different types of disfluencies serve different communicative functions? Based on a detailed qualitative analysis of the functions associated with each disfluency type, we organized our results into two disfluency groups: (1) Silent pauses, filled pauses, lengthenings, repetitions and discourse markers, and (2) Self-repairs, unrepaired disfluencies and disfluency comments.

#### 4.2.1 Silent and filled pauses, lengthenings, repetitions and disfluency markers

The first group comprises disfluency features that have been referred to frequently as fillers or placeholders in prior research (Amiridze et al., 2010). Similarities among these features have been noted in the literature. Stenström and Svartvik (1994) classified silent and filled pauses, repetitions, and verbal fillers within a single category, while Moniz et al. (2007) identified functional parallels between filled pauses and prolongations.

Although authors such as Crible et al. (2022) chose not to discriminate between fluent or disfluent uses of discourse features prior to the annotation, the objective of our study was to examine the disfluent end of the fluency spectrum. Accordingly, we introduced this distinction in the annotation of the data, following the instructions outlined in Table 5. Filled pauses and most repetitions are perceived consistently as disfluent, while silent pauses and lengthening are perceived as disfluent only in some usages. As the overview in Section 2.2 shows, there is a tradition of distinguishing between disfluent and fluent usages of silent pauses and lengthenings, and we have attempted to do so despite the apparent difficulty of establishing objective criteria. Discourse markers are a very complex and extensively researched linguistic feature, but, with regard to disfluencies, they are a borderline item, and our study includes only a small set of discourse markers, which are related to disfluencies the most tightly and were termed disfluency markers.

All the features in this category can be understood as forms of pausing or hesitation (Lickley, 2001) in speech. Pausing ranged from very short, as in certain filled pauses, to longer durations, such as when using a discourse marker or a repetition. Although in the annotation process we distinguished between fluent and disfluent realizations along a continuum, our analysis aimed to provide a unified account of the functions shared by all instances of silent pauses and lengthening—not only those annotated explicitly in the data. Our findings indicate that the speaker’s motivation for pausing can generally be categorized into two main types: (1) Pausing to allow time for cognitive processing before continuing, and (2) Pausing to signal a syntactic or semantic boundary in the speech, or to add emphasis. Both types have been observed previously in studies such as those conducted by Cenoz (1998) and Tottie (2019). While the first category is, typically, perceived as disfluent, the second is considered an integral component of fluent speech.

Both fluent and disfluent uses of silent pauses and lengthening, as well as the use of filled pauses, repetitions and discourse markers, represent manifestations of a single general underlying function: segmenting speech into cognitively manageable units, or what Kjellmer (2003) referred to as a “thought unit.” In his investigation of filled pauses, Kjellmer (2003, p. 174) observed: “One main function of er(m) thus seems to be to introduce what I will loosely call a new “thought unit,” a word, a phrase and sometimes a whole clause.” Our interpretation aligns with his. Such segmentation benefits both the speaker and the listener, as it facilitates speech production and comprehension.

By organizing speech into manageable units, these features not only segment the flow of speech, but, more importantly, connect these segments into a cohesive speech or, in other words, to establish fluency (Crible, 2018; Gotz, 2013; Moniz et al., 2007). The connective function, well-documented in the case of discourse markers, is just as critical as the segmentation function. In cases of disfluency, silent or filled pauses, lengthening, repetitions, discourse markers and clusters of these features (e.g., *[silentPause] [filledPause] [filledPause] i’d say)* help to ensure that the speech remains connected.

A range of additional, context-dependent functions may be attributed to these discourse features. Several scholars have emphasized their interpersonal roles (Kjellmer, 2003; Kosmala, 2024; Schegloff, 2010; Tottie, 2014, 2016, 2019). However, one of the few studies that distinguishes explicitly between own-communication management and interactive communication management functions of disfluencies (Kosmala, 2024, p. 125) reported that the proportion of own-communication management functions are “overwhelmingly greater.” Our interpretation is that interpersonal functions emerge only within specific situational contexts, and are not encoded inherently in a particular disfluency feature per se. Rather, they arise from the role that the feature plays within the broader discourse segment in which it occurs. Accordingly, we propose that such functions be regarded as strategic exploitations, rather than as part of their core functions, and we do not investigate these functions systematically.

In Appendix, we present four examples of speech from four different speakers in our data set. The first and second examples are from public speech, while the third and fourth are from private speech. Each example consists of a spoken utterance segmented into smaller units, based on the speaker’s use of silent and/or filled pauses, lengthenings, repetitions and discourse markers. It is evident that speech units between pauses typically range from two to four words in length, although single-word units are also frequent, as well as longer units of ten words or more.

Segmenting speech into cognitively manageable units and connecting these units represent general functions common to all disfluency features within this first group. However, each feature also exhibits specific characteristics that distinguish it from the others in the group. Drawing on our analysis, and supported by findings from previous research, these distinguishing characteristics are outlined below.

1. A filled pause functions as a signal, indicating that the speaker has something to say. This accounts for why scholars associate certain uses of filled pauses with functions such as planning, turn-taking, attention-getting, emphasis, or the initiation of an utterance (Clark, 2002; Kjellmer, 2003; Schegloff, 2010; Tottie, 2019).
2. A silent pause serves as a signal that the speaker is thinking. In fluent usage, it indicates the end of a thought, acts as a cue for turn-taking, or is used stylistically for emphasis. Compared to a filled pause, it generally conveys a more positive connotation. The existing research confirms predominant usage of silent pauses for segmentation purposes, as well as an error repair strategic device (Rühlemann et al., 2013; Stenström & Svartvik, 1994), with the former usage being less frequent (Gyarmathy, 2019). Furthermore, their usage for stylistic purposes was also recognized previously (Duez, 1982; Rühlemann et al., 2013).
3. Lengthening occurs typically before a silent pause, a filled pause, or a repetition, signaling a slowdown in speech tempo. Studies on the functions of lengthening are relatively scarce. Existing research confirms its role in gaining time and maintaining fluency (Moniz et al., 2007).
4. Repetition signals that the speaker intends to continue speaking. It serves as a cognitively undemanding method to hold the floor, and/or it gives the speaker time to engage in linguistic and/or cognitive planning (Rieger, 2003), while providing more time than a mere silent or filled pause, as repetitions “often include both filled and unfilled pauses and syllable lengthening” (Gráf & Huang, 2019, p. 234). In borderline cases, repetition may also emphasize a particular part of the speech (Rieger, 2003; Stenström & Svartvik, 1994), and/or ensure that the listener does not miss important information.
5. The disfluency markers included in our study represent a very limited subset of discourse markers that reflect speech production processes. In the literature they are often referred to as filler words (Laserna et al., 2014). In our data these expressions frequently include verbs such as *to say* (*bi rekel, bom rekel*), *to know* (*ne vem*), and *to think* (*mislim*). Diverse functions were assigned to these fillers, such as hedging, agreeing, and monitoring across various domains, including interpersonal, sequential, and rhetorical (Fu et al., 2024). Similar to repetition, they help the speaker hold the floor while providing more time to formulate their thoughts than a filled pause would.

#### 4.2.2 Self-repairs, unrepaired disfluencies, abandoned structures, and disfluency comments

While the previous group of disfluency features is associated with hesitation (Lickley, 2001), this group can be classified under self-repair (Lickley, 2001). Self-repairs have been explained in terms of speakers monitoring their own speech (Levelt, 1983) and pursuing the ideal delivery (Clark, 2002). Within the conversation analytic framework, in addition to “production errors,” “interactional errors” are identified, referring to attempts to speak appropriately to a co-participant or within a particular situational context (Jefferson, 1974).

Self-repairs have been studied extensively, while unrepaired disfluencies have received little scholarly attention, and abandoned structures and disfluency comments are not typically regarded as prototypical instances of disfluency. Given that these three phenomena are relatively marginal compared to self-repairs, yet share notable similarities, we have grouped them together. However, we caution readers that this decision is motivated partly by practical considerations, as important differences also exist between them.

In our data, these disfluency features are linked to three types of speech production processes: (1) The speaker adjusts or modifies the initiated structures to articulate the idea, termed as “appropriateness repair” by Lickley (2001), [2] The speaker identifies and corrects an error or inaccuracy, termed as “error repair” by Lickley (2001), and [3] The speaker experiences difficulty in articulating words or ideas fluently, typically manifesting in pronunciation-related self-repairs and complex self-repairs. In the case of self-repairs, the speaker makes corrections, whereas, in the case of unrepaired structures, the speaker does not deem it necessary to correct the disfluency, as the intended meaning is clear without correction. Table 7 provides a more detailed description of each disfluency type, along with examples from our data.

**Table 7.** Usages of self-Repairs, Unrepaired Disfluencies and Disfluency Comment.

Disfluency type	Usage	Example^a^	Translation of the example
Self-repair pronunciation	The speaker encounters difficulty in articulating a word fluently and corrects the pronunciation.	(1) *v razširjeni program se* ***uče()*** *[.]* ***učenci*** *vključujejo prostovoljno* (the sign [.] marks an interregnum point)	in the extended program **stu()** [.] **students** participate voluntarily (the sign [.] marks an interregnum point)
	The speaker identifies an error in pronunciation and corrects it.	(2) *in novinarske* ***nar()*** *[.]* ***organizacije*** *smo nekak podpirale to novo pravico*	and journalistic **nar()** [.] **organizations** somehow supported this new right
Self-repair lexicogrammar	The speaker modifies the structure for expressing an idea.	(3) *vendar njegova ljubezen nekako skozi* ***celotno eee*** *[.]* ***celoten*** *film ostaja neuresničena*	however, his love somehow throughout the **entired uh** [.] **entire** film remains unfulfilled
	The speaker expresses an idea with greater precision, or by providing additional information.	(4) *in tudi v tej borbi za oblast za moč za vladanje se je kazal eee to da nekdo ki ima izrazita* ***moralna načela ki eee*** *[.]* ***moralno-etična načela*** *eee zelo težko eee provzaprov ostane na oblasti*	and even in this struggle for power for control for governance it became evident uh that someone with strong **moral principles which uh** [.] **moral-ethical principles** uh finds it very difficult in fact to remain in power
	The speaker identifies a grammatical or semantic error and corrects it.	(5) *ker je zelo logično* ***da človek ne poškodu()*** *[.]* ***da robot ne poškoduje***	because it is very logical that **a human does not harm** [.] **that a robot does not harm**
Self-repair restart	The speaker abandons the already initiated structure and begins expressing the idea anew.	(6) *in eee bliže sem bil domu* ***bolj sem že tisto*** *[.]* ***bolj si že bil doma z mislimi*** *ne*	and uh the closer I was to home **the more I was that** [.] **the more I was at home in my thoughts** y’know
Self-repair complex	The speaker encounters difficulty in expressing an idea fluently, and makes multiple corrections to address the challenges.	(7) *eee seveda pa v bistvu* ***ne os() zapo()*** *[.]* ***zadolžitve ne ostani()*** *[.]* ***ne ostajajo***	uh of course actually **it don’t st() obri()** [.] **obligations don’t stan()** [.] **don’t stay**
Unrepaired structure	The speaker modifies the framework for articulating an idea, but this does not hinder understanding.	(8) *na eee ministrstvu za delo eee* ***je bil že pred časom eee sprejeta*** *eem možnost in pravica* (correct “je bila . . . sprejeta”)	uh the Ministry of Labor uh **is some time ago uh accepted** uh the option and the right (correct “has . . . accepted”)
	The speaker omits certain expected elements of the structure, but this does not hinder understanding.	(9) *mi imamo na primer že zdaj v našem eem eem pač zakonu o* ***avtorskih sorodnih*** *pravicah na primer pravico eem klipinga* (correct “avtorskih in sorodnih”)	we already have, for example, in our uh uh y’know law on **copyright related rights** the right to uh clipping for instance (correct “copyright and related rights”)

a All the examples can be accessed and listened to using the Gos concordancer at https://viri.cjvt.si/gos/ by searching for the following expressions: (1) “uklučujejo prostovoljno”; (2) “organizacije smo,” (3) “nekako skozi celotno”; (4) “moralna načela”; (5) “da robot ne”; (6) “bio doma”; (7) “zadolžitve ne”; (8) “je bil že pred časom”; (9) “avtorskih sorodnih”; (10) “tista vožnje”; (11) “vidla mezmes”; (12) “koča gor”; (13) “eksponentov”; (14) “pa ni mus”; (15) “gleat pa se tam.”

Blocks are infrequent in our data and are functionally most similar to unrepaired pronunciations. Similarly, infrequent are nonlinguistic sounds, which resemble disfluency comments functionally.

### 4.3 Contextual features

In this Section we tackle the third research question: Is there a meaningful relationship between physical, social, cognitive, and other factors that influence speakers’ communication behavior and the functions of disfluencies that might explain the quantitative differences between private and public speech? We first outline how the speakers in our data are influenced by the contextual features defined in Section 3.3, namely: (1) The scene; (2) The assigned relevance and importance; (3) The aims; (4) The conversational dynamics; (5) The positions and social relations; and (6) The social affiliations. We then discuss the potential impact of these features on the frequency of disfluency types. Although the interpretations presented here have been considered carefully and reflected upon critically throughout the course of the analysis, they remain our own interpretations of contextual factors, and are therefore inherently subjective to some extent.

#### 4.3.1 Analysis of context in public and private speech data

*Scene*. Our public data covers various settings: face-to-face events like press or scientific conferences, online events during COVID-19, live radio interviews, and parliamentary speeches broadcast on national television. The speakers often faced stress, managed differently, depending on their experience—lecturers, politicians, and journalists typically had established strategies, while less experienced speakers felt more pressure. Most spoke spontaneously, though some prepared in advance. Speakers in the media faced time constraints, typical for the media setting. Private speech recordings were conducted by students, often involving friends or relatives in quiet home settings. The speakers discussed topics like movies, books, travel, or hobbies, or engaged in conversations with friends or relatives. Handheld recorders and personal microphones were used for recording. While the home environment helped some speakers feel at ease, others felt pressured by the recording’s formality, or struggled with the topics. The recordings were typically unscripted. There were no time constraints; on the contrary, the speakers were encouraged to deliver extended speech, lasting up to 30 min.

*Attributed relevance and importance*. Public speech carries high relevance and expectations for perfect delivery, with the language expected to be standard, formal, and fluent. It is expected to carry significant informational value. In private speech, the awareness that recordings were for research likely made speakers place more importance on their speech than they would in casual conversations. However, the assigned relevance and importance were lower than in public speech and the expected informational value was low.

*Aims*. In public speech, the primary goal is to convey information or opinions important for society, with the speakers feeling a responsibility to deliver content appropriately. The data set excludes informal or entertaining content. In private speech no specific tasks or objectives were set, and the participants were free to choose topics, aiming for casual and natural conversation. However, the awareness of being recorded may have influenced their behavior and speech flow subtly.

*Conversational dynamics*. In public speech, 16 out of the 29 samples (55%) were monologs, while 13 were dialogs, with turns in the dialogs averaging three times longer than in the private conversations. In private speech, 14 out of the 21 events (67%) were monologs about personal topics, and the remaining seven were dialogs with friends, featuring significantly shorter turns compared to the public speech dialogs.

*Positions and social relations*. In public speech, most speakers hold positions of authority or expertise and address a formal audience, often under pressure to uphold their status. In dialogs, the interviewers guided the conversation, while the interviewees provided information and demonstrated knowledge. In private speech, the recordings occurred in the speakers’ homes, with only friends or relatives present, fostering personal and informal relationships.

*Social affiliations*. The public speakers include academics, well-known experts, elected politicians in the parliamentary data, and professional journalists, all highly educated. They represent diverse professions, including Information Technology, Medicine, Economics, Social work, Linguistics, Music, History, and more. The private speakers comprised both genders, various educational backgrounds and a wide range of age groups. They came from all regions of Slovenia, representing both urban and rural areas. All the private speakers were native Slovene speakers who used their natural language style during the recordings. Table 8 provides a summary of the contextual features in public and private speech, focusing on aspects that could potentially influence the use of disfluencies.

**Table 8.** Summary of Contextual Features in Public and Private Speech Data.

Contextual feature	Public speech data	Private speech data
Scene	The speakers are under emotional stress	The speakers experience very low levels of emotional stress
	Time constraints	Unlimited time
	Often partially pre-scripted speech	Unprepared speech
Attributed relevance and importance	Very high relevance and expectations for perfect delivery	Middle relevance, expectations for delivery were similar or slightly higher than in casual conversation
Aims	To convey information or opinions important for society	No specific tasks or objectives were set
Conversational Dynamics	55% monologs; dialogs with turns 3 times longer than those in private speech	67% monologs; dialogs with turns 3 times shorter than those in public speech
Positions and social relations	Formal relationship	Personal and informal relationships
	Pressure to uphold the status of authority or expertise	No specific pressure regarding the social position
Social affiliations	Highly educated individuals and/or experienced public speakers	Everyday individuals

#### 4.3.2 The effects of contextual features on the frequency of disfluencies

In Section 4.1 we measured that the disfluencies were 1.22 times more likely to occur in private speech data compared to public speech data. In Section 4.2, we identified that disfluencies generally serve two general functions: (1) Some disfluency types are used primarily to segment speech into cognitively manageable units, and, at the same time, serve as connectors between these units; (2) Other disfluency types refer to problems of articulation of words or ideas, adaptations or changes in articulation/already started structures, or error correction.

A critical examination of the contextual features suggested several reasons for the lower frequency of disfluencies in public speech in general. First, the high relevance assigned to public speech, coupled with expectations for perfect delivery, formal relationships, and the goal of conveying information or opinions important to society, likely promotes a formal speech style with fewer disfluencies. The authors (Bortfeld et al., 2001) suggested that speakers reduce disfluencies effectively when circumstances demand it, a finding supported by our analysis. Second, public speech is often partially pre-scripted, a feature previously shown to reduce disfluency use (Tonetti Tübben & Landert, 2022). Third, speakers in public speech face greater time constraints compared to those in private speech. It has been suggested (Moniz et al., 2014) that reduced time pressure allows speakers more freedom to adapt and modify initiated structures, contributing to higher disfluency rates in private speech. Finally, highly educated individuals, including many of the experienced public speakers in our data set, likely possess better-developed skills for minimizing disfluencies compared to the general population.

Next, the quantitative analysis revealed that not all disfluency types follow the general trend of being more frequent in private speech. Filled pauses, unrepaired pronunciations and blocks deviated from this pattern, more likely occurring 1.3 times in public speech compared to private speech. In Section 4.2, we established that filled pauses function as signals indicating the speaker has something to say. Their higher frequency in public speech may be linked to conversational dynamics, as public speech features dialog turns three times longer than those in private speech, creating a greater need to signal that the speaker has not finished. In addition, the use of filled pauses may be influenced by time constraints and emotional stress in public settings, as they may be perceived as more convenient and shorter pausing devices compared to other time-gaining strategies, such as silent pauses, repetitions, or disfluency markers.

Unrepaired pronunciations and blocks were also more frequent in public speech compared to private speech. In unrepaired pronunciations, the speech is fast or unclear, but does not hinder understanding. Blocks serve a similar function. Although not statistically significant due to their low frequency, it was also notable that the speakers used more self-repair pronunciations in public speech than in private speech. All this suggests that speakers generally experience more pronunciation issues in public speech. The analysis of contextual features revealed that speakers face significantly higher emotional stress in public than in private speech, and much higher expected informational value. All this imposes a cognitive burden, and, while the public speakers in our data managed this burden well at the level of lexical expression and utterance structure, it manifested at the level of articulation. In addition, the time constraints in public speech may compel speakers to continue speaking when their pronunciation is understandable, even if not perfect, rather than taking the time to make a self-repair.

## 5 Discussion

We have investigated how disfluency usage differs between public and private speech. In our first research question, we investigated whether there was any regularity in how speakers used different types of disfluencies. To answer this question we collected balanced data, 29 samples with 47 speakers, totaling 23,000 words for public speech, and 28 samples with 28 speakers totaling 15,000 words for private speech. We developed our own disfluency annotation scheme to annotate the data. The scheme was corpus-driven, and aimed to be simple and robust. We distinguished between vocal and verbal disfluencies at the first level, a distinction also made by Kosmala (2024), building on the work of Guaïtella (1993) and others. At the second level, we distinguished the basic disfluency types in each group, including all the typical disfluency types (filled pauses, repetitions, self-repairs), atypical, but common disfluency types (silent pauses, lengthenings, discourse/disfluency markers, editing terms/disfluency comments), and rarely annotated disfluency types (block, abandoned/incomplete utterance). Self-repairs were categorized into several subtypes, all of which have been noted previously in the literature. Self-repaired pronunciation is referred to in the literature as an articulation error or slip of the tongue, while complex self-repair aligns with the concept of complex disfluency, and self-repaired restarts are typically described as restarts. Self-repaired lexicogrammar encompasses syntactic, morphological and lexical repairs, as we found it challenging to differentiate reliably between these established types. A new category is unrepaired disfluencies. We annotated a total of 155 instances of unrepaired disfluencies (80 pronunciations and 75 structures), suggesting that this is not a rare feature and deserves scholarly attention. The quantitative results showed that disfluencies, in general, have a positive association with private speech, with an odds ratio of 1.22. However, not all disfluency types followed this trend. Filled pauses, unrepaired pronunciations, and blocks were more frequent in public speech, with an odds ratio of 1.3.

To investigate the use of disfluencies in more detail, we asked second whether different types of disfluencies serve different communicative functions. We defined two general functions: (1) Some disfluency types are used primarily to segment speech into cognitively manageable units, and, at the same time, serve as connectors between these units; (2) Other disfluency types refer to problems of articulation of words or ideas, adaptations or changes in articulation/already started structures, or error correction. A range of additional, context-dependent functions—such as interpersonal and communicative functions—may be attributed to each of these discourse features, depending on the particular context of use. We therefore propose distinguishing the most general and typical functions as the focus of our analysis, while acknowledging that less frequent, context-sensitive exploitations also exist and merit further investigation. Our analyses showed that most vocal disfluencies served the first function, while most verbal disfluencies served the second function. However, the overlap is not absolute; blocks are exceptions among vocal disfluencies, and repetitions and disfluency markers are exceptions among the verbal disfluencies. Furthermore, we found that each annotated disfluency type has its own specific characteristics and patterns of use. We have described these, thus justifying the defined disfluency annotation scheme, and confirming that none of the disfluency types is an arbitrary speech feature, but has its own specificities, and can serve the listener as well as the speaker. Some of the identified characteristics confirm that disfluencies are not only a hesitation and self-repair mechanism, but, at the same time, are often useful signals for turn-taking (especially vocal disfluencies), a stylistic feature (e.g., repetition, silent pause), or metainformation to the listener that the speaker is trying to express information in an accurate and comprehensible way.

Third, we sought a meaningful explanation for the relationship between contextual features in public and private speech, and the functions of disfluencies that could account for the differences in disfluency frequencies in the two settings. We conducted a detailed analysis of contextual features for both public and private data. One cannot explain the quantitative results reliably without knowing the exact circumstances in which the speakers were recorded. It is not the fact of speaking in front of a public/broadcasting per se which influences the use of disfluencies, but particular contextual features need to be taken into account, to understand what motivates speakers to use particular disfluencies. In our case, these contextual features were the scene, the ascribed relevance and importance, the goals and the interactional dynamics. Our conclusions were that the speakers reduce disfluencies in public speech due to its high relevance and formal expectations, partial pre-scripting, time constraints, and the advanced skills of highly educated or experienced speakers. The opposite trend, more frequent use of filled pauses in public speech, is likely due to the longer dialog turns requiring signals of continuity. Furthermore, time constraints and emotional stress in public speech make filled pauses a more practical and efficient pausing strategy compared to silent pauses, disfluency markers, or repetitions. The higher frequency of unrepaired pronunciations and blocks in public speech reflects a broader trend of speakers encountering more pronunciation issues in public speech. We attributed this to emotional stress, the anticipated high informational value, and time constraints in public speaking. This leads speakers to prioritize continuing their speech when their pronunciation is understandable, even if not perfect, rather than taking the time to make a self-repair.

It is important to acknowledge the limitations of this study. First, the speakers in the public and private speech samples were not the same, which may have influenced the quantitative results, as disfluency patterns can be speaker-specific to some extent. Nevertheless, we consider it more valuable to analyze the speech of real-life public speakers—even if their private speech is unavailable—than to rely on the seemingly public speech of less experienced speakers, such as students. We also included a relatively large sample of speakers, to mitigate the influence of individual speaking styles. Second, the analyses of disfluency functions, contextual features, and their potential impact on the frequency of disfluency types are qualitative in nature and based on our interpretations. As such, they inevitably involve a degree of subjectivity, and should be taken with caution.

## 6 Conclusion

This study explored how disfluency usage varies between public and private speech settings, focusing on three aspects: the frequency of disfluencies, their communicative functions, and the relationship between contextual features and disfluency usage. Our findings demonstrated that public speech exhibits fewer overall disfluencies than private speech, except for filled pauses, unrepaired disfluencies and blocks, which are more frequent in public contexts. Our interpretations in the qualitative analysis showed that disfluencies can be grouped into two basic functions. Silent and filled pauses, lengthenings, repetitions and disfluency markers are used primarily to segment speech into cognitively manageable units, and, at the same time, serve as connectors between these units. Self-repairs, unrepaired disfluencies and disfluency comments refer to problems of articulation of words or ideas, adaptations or changes in articulation/already started structures, or error correction. In the final step of our analysis, we argued that all frequency trends can be explained meaningfully by considering the detailed physical, social, cognitive, and other contextual factors of the recorded situations which influence the speakers’ communication behavior systematically. Although the results should be interpreted with caution due to the interpretative nature of the qualitative analysis and the potential confounding effect arising from the involvement of different speakers in the public and private speech samples, we have sought to mitigate these limitations by employing a large, balanced sample of speakers in authentic communicative contexts, and by reflecting on our interpretations critically, grounding them in relevant findings from previous scholarly work.

This study broadens our understanding of how speakers adapt their speech production strategies to different interactional contexts through their use of disfluencies. Unlike earlier studies, we employed a balanced data set spanning diverse genres and a wide range of speakers, combining quantitative and qualitative analyses. Our approach included a corpus-driven categorization of disfluencies, ensuring that the categories reflect authentic speech patterns rather than preconceived theoretical assumptions. We adopted a systematic approach, not only to identify and confirm the differences in disfluency usage, but also by trying to explain their underlying causes. By focusing on Slovenian, we expanded the existing studies to a new linguistic context, and helped ensure that findings in the field are not overly dependent on results from a single or a few most-researched languages. However, like any study, ours also has its limitations. Although the data set is balanced, it represents a small sample of language use. Public speech samples are restricted to formal events, excluding entertaining public speech, while private speech features a high proportion of monologs and lacks more interactive data. The data set is limited to native Slovene speakers, requiring caution when generalizing the findings to other languages and cultures. The qualitative analyses relied on researcher interpretation, which, despite a rigorous methodology, retains a degree of subjectivity. Other variables, such as spontaneity or interactivity, may intersect with the studied variable, warranting further exploration. Future research is needed to address these limitations and expand on our findings. Despite these limitations, this study provides valuable insights into the influence of interactional context on disfluency use, offering a foundation for future cross-linguistic and context-based research on speech production strategies.

## Appendix

### Examples from our data

All the examples can be accessed and listened to via the concordancer of the Slovenian reference corpus GOS (https://viri.cjvt.si/gos/), using the search phrase provided for each example. There was no turn-taking in the cited examples.

**Example 1.** (search “in tu se navezujem” and “še čez eno mejo”)

A professional discussion on the topic of spoken Slovenian in the media. The speaker is a lecturer and an experienced public speaker. He explains the beginnings of his career.

**Table:**

Uninterrupted unit of speech	English translation	Annotated disfluency at the end of a unit	Not annotated, but notable at the end of a unit
kasneje m() pa me je pot potem	uh later the path		lengthening
vodila	led me		lengthening; silent pause
predvsem čez mejo	across the border		silent pause
in tu se navezujem na	and here I connect to	silent pause	lengthening
misli eee	the thoughts uh	filled pause	lengthening; silent pause
direktorja	of the director	silent pause	lengthening
ZRC SAZU	of ZRC SAZU		lengthening; silent pause
čez mejo	across the border		lengthening; silent pause
in še čez eno mejo	and across another border	silent pause	
v Prago	to Prague		silent pause

**Example 2.** (search “notranjimi pravili igre” and “poštenega in primernega”)

Public round table about journalists and copyrights. The speech of this speaker is a longer talk, and addresses the importance of collective management and implementation of the new publishers’ related rights under EU Copyright Directives.

**Table:**

Uninterrupted unit of speech	English translation	Annotated disfluency at the end of a unit	Not annotated, but notable at the end of a unit
ker	because	lengthening	silent pause
samo skupej e	only together uh	lengthening; filled pause	silent pause
in	and		
z temi e	with these uh	lengthening; filled pause	silent pause
notranjimi pravili igre	internal rules of the game		silent pause
ki jih imajo kolektivne	that collective	lengthening	
organizacije regulirane	organizations have regulated		silent pause
zakonsko določene e	legally defined uh	filled pause	lengthening; silent pause
smo prepričani da bi lahko pršli do nekega eem eem	we are convinced that we could reach some sort of uh	lengthening; filled pause; silent pause; filled pause	silent pause
poštenega in primernega nadomestila	fair and appropriate compensation	lengthening	silent pause
tud	even		silent pause
do tega končnega e	for this final uh	lengthening; filled pause	silent pause
člena v verigi ki je	link in the chain which is		lengthening; silent pause
avtor	the author		silent pause

**Example 3.** (search “tam nekaj pregradili”)

Conversation at home between a husband and wife. The husband asks questions most of the time, in example 3 about the situation in a hospital were his wife works.

**Table:**

Uninterrupted unit of speech	English translation	Annotated disfluency at the end of a unit	Not annotated, but notable at the end of a unit
kaj pa tisto zaj ko so	what about now when they	repetition	lengthening; silent pause
ko so rekli da vam bojo	said they would	lengthening	silent pause
tam nekaj pregradili kaj bote zaj meli oni	build something there will you now have that	silent pause	
kovid	COVID	silent pause	
oddelek?	department?		end of turn

**Example 4.** (search “gradbišč dal skoz”)

A conversation at home between a husband and wife, discussing the husband’s work on a construction site.

**Table:**

Uninterrupted unit of speech	English translation	Annotated disfluency at the end of a unit	Not annotated, but notable at the end of a unit
jaz pa mislim da	I think that	lengthening	
si že tolko gradbišč dal skoz	you’ve already been through so many construction sites	silent pause	lengthening
da še boš to tudi veš	that this one you’ll also y’know	disfluency marker	silent pause
obvladal	handle		

## References

[bibr1-00238309251380518] Amiridze N. Davis B. Maclagan M. (Eds.). (2010). Fillers, Pauses and Placeholders. Typological Studies in Language (Vol. 93). John Benjamins Publishing Company. http://www.jbe-platform.com/content/books/9789027287762

[bibr2-00238309251380518] Besser J. Alexandersson J. (2007). A comprehensive disfluency model for multi-party interaction. In Bunt H. Keizer S. Paek T. (Eds.), Presented at the SIGDIAL 2007 Proceedings of the 8th SIGdial workshop on discourse and dialogue (pp. 182–189). Association for Computational Linguistics. https://aclanthology.org/2007.sigdial-1.31

[bibr3-00238309251380518] Bortfeld H. Leon S. D. Bloom J. E. Schober M. F. Brennan S. E. (2001). Disfluency rates in conversation: Effects of age, relationship, topic, role, and gender. Language and Speech, 44(2), 123–147. 11575901 10.1177/00238309010440020101

[bibr4-00238309251380518] Brennan S. E. Schober M. F. (2001). How listeners compensate for disfluencies in spontaneous speech. Journal of Memory and Language, 44(2), 274–296.

[bibr5-00238309251380518] Cenoz J. (1998). ERIC ED426630: Pauses and communication strategies in second language speech. Retrieved from https://archive.org/details/ERIC_ED426630

[bibr6-00238309251380518] Clark H. H. (2002). Speaking in time. Speech Communication. ESCA Workshop on Dialogue and Prosody, 36(1), 5–13.

[bibr7-00238309251380518] Corley M. MacGregor L. J. Donaldson D. I. (2007). It’s the way that you, er, say it: Hesitations in speech affect language comprehension. Cognition, 105(3), 658–668. 17173887 10.1016/j.cognition.2006.10.010

[bibr8-00238309251380518] Crible L. (2018). Discourse markers and (dis)fluency: Forms and functions across languages and registers. Pragmatics & beyond new series (Vol. 286). John Benjamins Publishing Company. http://www.jbe-platform.com/content/books/9789027264305

[bibr9-00238309251380518] Crible L. Degand L. (2019). Domains and functions: A two-dimensional account of discourse markers. Discours. Revue de linguistique, psycholinguistique et informatique. A Journal of Linguistics, Psycholinguistics and Computational Linguistics, 24.

[bibr10-00238309251380518] Crible L. Didirková I. Dodane C. Kosmala L. (2022). Towards an inclusive system for the annotation of (dis)fluency in typical and atypical speech. Clinical Linguistics & Phonetics, 38, 381–398. 10.1080/02699206.2022.2126330 36205188

[bibr11-00238309251380518] Duez D. (1982). Silent and non-silent pauses in three speech styles. Language and Speech, 25(1), 11–28.

[bibr12-00238309251380518] Engelhardt P. E. Markostamou I. (2024). Disfluency across the lifespan: An individual differences investigation. Aging, Neuropsychology, and Cognition, 32, 1–25. 10.1080/13825585.2024.2354958 38767398

[bibr13-00238309251380518] Fox Tree J. E . (1995). The effects of false starts and repetitions on the processing of subsequent words in spontaneous speech. Journal of Memory and Language, 34(6), 709–738.

[bibr14-00238309251380518] Fox Tree J. E . (2001). Listeners’ uses of um and uh in speech comprehension. Memory & Cognition, 29(2), 320–326. 11352215 10.3758/bf03194926

[bibr15-00238309251380518] Fraundorf S. H. Watson D. G. (2011). The disfluent discourse: Effects of filled pauses on recall. Journal of Memory and Language, 65(2), 161–175. 21765590 10.1016/j.jml.2011.03.004 PMC3134332

[bibr16-00238309251380518] Frobenius M. (2022). A pragmatic approach to fluency and disfluency in learner language: Cofluencies as sites of accountability, sequentiality, and multimodality. Pragmatics & beyond new series (Vol. 332). John Benjamins Publishing Company. 10.1075/pbns.332

[bibr17-00238309251380518] Fu Y. Afzaal M. El-Dakhs D. A. S. (2024). Investigating discourse markers “you know” and “I mean” in mediatized English political interviews: A corpus-based comparative study. Frontiers in Communication, 9, 1427062.

[bibr18-00238309251380518] Gotz S. (2013). Fluency in Native and Nonnative English Speech. John Benjamins Publishing Company. https://benjamins.com/catalog/scl.53

[bibr19-00238309251380518] Gráf T. Huang L. (2019). Repeats in native and learner English. In Fluency and disfluency across languages and language varieties (pp. 219–241). Presses universitaires de Louvain.

[bibr20-00238309251380518] Guaïtella I. (1993). Functional, acoustical and perceptual analysis of vocal hesitations in spontaneous speech [Working papers]., Department of Linguistics and Phonetics, Lund University.

[bibr21-00238309251380518] Gyarmathy D. (2019). The functions of silent pauses in spontaneous Hungarian speech. https://api.semanticscholar.org/CorpusID:261053367

[bibr22-00238309251380518] Hanks P. (2013). Lexical analysis: Norms and exploitations. The MIT Press. https://direct.mit.edu/books/book/2981/Lexical-AnalysisNorms-and-Exploitations

[bibr23-00238309251380518] Horvath V. (2007). Are there gender-based differences in disfluency phenomena? Or, are there “male” and “female” disfluencies in spontaneous speech? Magyar Nyelvor, 3(131), 315–323.

[bibr24-00238309251380518] Jefferson G. (1974). Error correction as an interactional resource. Language in Society, 3(2), 181–199.

[bibr25-00238309251380518] Kjellmer G. (2003). Hesitation. In defence of ER and ERM. English Studies, 84(2), 170–198.

[bibr26-00238309251380518] Kosmala L. (2024). Beyond Disfluency: The interplay of speech, gesture, and interaction. John Benjamins. https://www.jbe-platform.com/content/books/9789027247261

[bibr27-00238309251380518] Kosmala L. Morgenstern A. (2019). Should “uh” and “um” be categorized as markers of disfluency? The use of fillers in a challenging conversational context. In Fluency and disfluency across languages and language varieties. Presses Universitaires de Louvain.

[bibr28-00238309251380518] Laserna C. M. Seih Y.-T. Pennebaker J. W. (2014). Um . . . who like says you know: Filler word use as a function of age, gender, and personality. Journal of Language and Social Psychology, 33(3), 328–338.

[bibr29-00238309251380518] Levelt W. (1983). Monitoring and self-repair in speech. Cognition, 14(1), 41–104. 6685011 10.1016/0010-0277(83)90026-4

[bibr30-00238309251380518] Lickley R. J. (2001). Dialogue moves and disfluency rates. Workshop on Disfluency in Spontaneous Speech. https://api.semanticscholar.org/CorpusID:93842

[bibr31-00238309251380518] MacGregor L. J. Corley M. Donaldson D. I. (2010). Listening to the sound of silence: Disfluent silent pauses in speech have consequences for listeners. Neuropsychologia, 48(14), 3982–3992. 10.1016/j.neuropsychologia.2010.09.024 20950633

[bibr32-00238309251380518] Maclay H. Osgood C. E. (1959). Hesitation phenomena in spontaneous English speech. Word, 15, 19–44. https://www.scirp.org/reference/referencespapers?referenceid=3101186

[bibr33-00238309251380518] McDougall K. Duckworth M. (2017). Profiling fluency: An analysis of individual variation in disfluencies in adult males. Speech Communication, 95, 16–27.

[bibr34-00238309251380518] Meteer M. Taylor A. (1995). Dysfluency annotation stylebook for the switchboard corpus. http://cs.brandeis.edu/~cs140b/CS140b_docs/DysfluencyGuide.pdf

[bibr35-00238309251380518] Moniz H. Batista F. Mata A. I. Trancoso I. (2014). Speaking style effects in the production of disfluencies. Speech Communication, 65, 20–35.

[bibr36-00238309251380518] Moniz H. Mata A. I. Viana M. C. (2007). On filled-pauses and prolongations in European Portuguese. In Presented at the Interspeech 2007 (pp. 2645–2648). ISCA. https://www.isca-archive.org/interspeech_2007/moniz07_interspeech.html

[bibr37-00238309251380518] Oviatt S. (1994). Predicting and managing spoken disfluencies during human-computer interaction. Proceedings of the workshop on Human Language Technology—HLT ’ 94 (p. 222). Association for Computational Linguistics. http://portal.acm.org/citation.cfm?doid=1075812.1075859

[bibr38-00238309251380518] Passali T. Mavropoulos T. Tsoumakas G. Meditskos G. Vrochidis S. (2024). Artificial disfluency detection, uh no, disfluency generation for the masses. Computer Speech & Language, 89, 101711.

[bibr39-00238309251380518] Penttilä N. Korpijaakko-Huuhka A.-M. Kent R. D. (2019). Disfluency clusters in speakers with and without neurogenic stuttering following traumatic brain injury. Journal of Fluency Disorders, 59, 33–51. 10.1016/j.jfludis.2019.01.001 30641458

[bibr40-00238309251380518] Rieger C. L. (2003). Repetitions as self-repair strategies in English and German conversations. Journal of Pragmatics, 35(1), 47–69.

[bibr41-00238309251380518] Rühlemann C. Bagoutdinov A. O’Donnell M. B. (2013). Windows on the mind: Pauses in conversational narrative. International Journal of Corpus Linguistics, 16(2), 198–230.

[bibr42-00238309251380518] Schachter S. Christenfeld N. Ravina B. Bilous F. (1991). Speech disfluency and the structure of knowledge. Journal of Personality and Social Psychology, 60(3), 362–367.

[bibr43-00238309251380518] Schegloff E. A. (2010). Some other “Uh(m)”s1. Discourse Processes, 47(2), 130–174.

[bibr44-00238309251380518] Schmidt T. Wörner K. (2014). EXMARaLDA. In Handbook on corpus phonology (pp. 402–419). Oxford University Press.

[bibr45-00238309251380518] Sen P. (2020). Speech disfluencies occur at higher perplexities. In Zock M. Chersoni E. Lenci A. Santus E. (Eds.), Proceedings of the Workshop on the Cognitive Aspects of the Lexicon (pp. 92–97). Association for Computational Linguistics. https://aclanthology.org/2020.cogalex-1.11

[bibr46-00238309251380518] Shriberg E. (1994). Preliminaries to a theory of speech disfluencies [Doctoral dissertation]. University of California, Berkeley.

[bibr47-00238309251380518] Shriberg E. (2001). To “errrr” is human: Ecology and acoustics of speech disfluencies. Journal of the International Phonetic Association, 31(1), 153–169.

[bibr48-00238309251380518] Stenström A.-B. Svartvik J. (1994). Imparsable speech: Repeats and other nonfluencies in spoken English. In Oostdijk N. de Haan P. (Eds.), Corpus-based research into language, language and computers (pp. 241–254). Brill.

[bibr49-00238309251380518] Strassel S. (2003). Simple metadata annotation specification (Version 5.0 – 5/15/2003). Linguistic Data Consortium.

[bibr50-00238309251380518] Taschenberger L. Tuomainen O. Hazan V. (2019). Disfluencies in spontaneous speech in easy and adverse communicative situations: The effect of age. In Proceedings of DiSS 2019, Presented at the 9th Workshop on Disfluency in Spontaneous Speech (pp. 55–58). ELTE Faculty of Humanities. http://hdl.handle.net/10831/43593

[bibr51-00238309251380518] Taylor A. Marcus M. Santorini B. (2003). The penn treebank: An overview. In Abeille A. (Ed.), Treebanks: Building and using parsed corpora (pp. 5–22). Springer.

[bibr52-00238309251380518] Taylor T. J. (1997). Theorizing language. Pergamon.

[bibr53-00238309251380518] Tognini-Bonelli E. (2001). Corpus Linguistics at Work. John Benjamins. https://www.jbe-platform.com/content/books/9789027285447

[bibr54-00238309251380518] Tonetti Tübben I. Landert D . (2022). Uh and Um as pragmatic markers in dialogues: A contrastive perspective on the functions of planners in fiction and conversation. Contrastive Pragmatics, 4(2), 350–381.

[bibr55-00238309251380518] Toomaneejinda A. Saengboon S. (2022). Interactional sociolinguistics: The theoretical framework and methodological approach to ELF interaction research. LEARN Journal: Language Education and Acquisition Research Network, 15(1), 156–179.

[bibr56-00238309251380518] Tottie G. (2014). On the use of uh and um in American English. Functions of Language, 21(1), 6–29.

[bibr57-00238309251380518] Tottie G. (2016). Planning what to say: Uh and um among the pragmatic markers. In Kaltenböck G. Keizer E. Lohmann A. (Eds.), Studies in language companion series (Vol. 178, pp. 97–122). John Benjamins Publishing Company. https://benjamins.com/catalog/slcs.178.04tot

[bibr58-00238309251380518] Tottie G. (2019). From pause to word: Uh, um and er in written American English. English Language and Linguistics, 23(1), 105–130.

[bibr59-00238309251380518] van Dijk T. A . (1997). Cognitive context models and discourse. In Stamenov M. I. (Ed.), Advances in Consciousness Research (Vol. 12, p. 189). John Benjamins Publishing Company. https://benjamins.com/catalog/aicr.12.09dij

[bibr60-00238309251380518] van Dijk T. A . (2001). Discourse, ideology and context. Folia Linguistica, 30(1–2), 11–40.

[bibr61-00238309251380518] van Dijk T. A . (2007). Discourse, context and cognition. Discourse Studies, 8(1), 159–177.

[bibr62-00238309251380518] Verdonik D. (2006). Popravljanja v spontano tvorjenih izjavah. Slavistična revija, 54(2), 188–203.

[bibr63-00238309251380518] Verdonik D. Bizjak A. Žgank A. Maučec M. S. Trojar M. Gros J. Ž. Dobrišek S. (2024b). Strategies for managing time and costs in speech corpus creation: Insights from the Slovenian ARTUR corpus. Language Resources and Evaluation, 59, 891–911. https://link.springer.com/article/10.1007/s10579-024-09746-8#article-info

[bibr64-00238309251380518] Verdonik D. Dobrovoljc K. Erjavec T. Ljubešić N. (2024a). Gos 2: A new reference corpus of spoken Slovenian. In Proceedings of the 2024 Joint International Conference on Computational Linguistics, Language Resources and Evaluation (LREC-COLING 2024) (pp. 7825–7830). https://aclanthology.org/2024.lrec-main.691/

